# Changes in the trajectories of drug-free friendships and substance use among a cohort of individuals with multiple substance use disorders

**DOI:** 10.1177/14550725251332929

**Published:** 2025-04-16

**Authors:** Fredrik D. Moe, Tore Tjora, Christian Moltu, James R. McKay, Egon Hagen, Aleksander Erga, Jone Bjornestad

**Affiliations:** Department of Social Studies, Faculty of Social Sciences, 56627 University of Stavanger, Stavanger, Norway;; Centre for Alcohol and Drug Research, 60496 Stavanger University Hospital, Stavanger, Norway; Department of Social Studies, Faculty of Social Sciences, 56627 University of Stavanger, Stavanger, Norway; Department of Psychiatry, District General Hospital of Førde, Førde, Norway; Centre for Alcohol and Drug Research, 60496 Stavanger University Hospital, Stavanger, Norway;; Department of Psychiatry, Perelman School of Medicine, University of Pennsylvania, Philadelphia, PA, USA;; Philadelphia VA Medical Center, Philadelphia, PA, USA; Centre for Alcohol and Drug Research, 60496 Stavanger University Hospital, Stavanger, Norway; Centre for Alcohol and Drug Research, 60496 Stavanger University Hospital, Stavanger, Norway;; The Norwegian Centre for Movement Disorders, 60496 Stavanger University Hospital, Stavanger, Norway;; Department of Biological and Medical Psychology, Faculty of Psychology, University of Bergen, Bergen, Norway; Department of Social Studies, Faculty of Social Sciences, 56627 University of Stavanger, Stavanger, Norway;; Department of Psychiatry, District General Hospital of Førde, Førde, Norway;; TIPS – Network for Clinical Research in Psychosis, 60496 Stavanger University Hospital, Stavanger, Norway

**Keywords:** debut age, drug-free friendships, gender, social support, SUD recovery

## Abstract

**Aims:** We used reports (*n* = 208) of drug-free friendships and alcohol and drug use by people diagnosed with substance use disorder in order to investigate their annual change trajectories across 4 years after treatment and the association between these trajectories and debut age and gender. **Methods:** The participants were recruited from the Stavanger region, Norway. Using cross-sectional analysis, we first examined the relationship between “alcohol and drug use” and “drug-free friendships” across the five follow-ups. We tested whether these associations were significant using chisquare chi-squared tests. Second, we developed three latent growth curve models examining the association between “alcohol and drug use” and “drug-free friendships”. **Results:** Our analysis displays a stable drug-free friendships pattern across follow-ups. Only in the fourth follow-up was there a significant association between lower “alcohol and drug use” and having “drug-free friendships” (χ^2^ = 18.27, df = 8, *p* < .05). In model 1, we found no association between gender, debut age, and alcohol and drug use; model 2 had significant variance on intercept but not on slope; model 3 had good fit (χ^2^ = 44.33, df = 39, comparative fit index = 0.98, root mean square error of approximation = 0.027). However, we did not find any significant regression path between the “alcohol and drug use” and “drug-free friendships” slopes. **Conclusions:** Drug-free relationships were in the studied cohort group found to have little influence on reducing alcohol and drug use, while debut age and gender were unrelated to use trajectories across 4 years. We suggest that future research should focus on the frequency and quality of drug-free friendships and participants’ friendship assessments because previous research has found such relationships to facilitate recovery.

## Introduction

Social support that facilitates healthy community belonging is essential to obtaining and maintaining recovery from substance use disorder (SUD) (Vigdal et al., 2022). Recovery from SUD is considered a protracted, multidimensional, heterogeneous process (Witkiewitz et al., 2020). The clinical recovery framework depicts SUD as a mental disorder containing specific core symptoms (Liberman et al., 2002). The symptom criteria are based on researcher-derived thresholds and predefined objectives, and recovery is achieved when symptoms subside below a diagnostic threshold. Within a personal recovery tradition, recovery is viewed as going beyond a reduction in core symptoms, and the focus is rather on contributory citizenship, identity change, connectedness, hope, optimism, meaning in life and the ability to build a new life despite the presence of symptoms (Davidson et al., 2007; Leamy et al., 2011).

Both the clinical and personal recovery frameworks have been criticised for overemphasising intrapersonal experiences and underestimating the interpersonal context of recovery (Price-Robertson et al., 2017). It is, however, currently common across traditions to consider symptom reduction as expedient to obtaining and maintaining recovery. Further, the focus on interpersonal aspects has gradually received more general support (Fowler et al., 2019; Pettersen et al., 2019).

Polysubstance disorder is common in clinical samples, although it is not a specified diagnosis in the Diagnostic and Statistical Manual of Mental Disorders, 5th edition (DSM-5) (Bhalla et al., 2017; Hasin et al., 2013). Among patients seeking treatment for mono-substance use disorder polysubstance use is frequent (Hetland et al., 2021; Onyeka et al., 2012). It is important to address polysubstance use since it seems to reflect the reality in treatment situations. The current study is part of the Norwegian Stavanger Study of Trajectories in Addiction (STAYER), a prospective naturalistic follow-up study of change trajectories among people diagnosed with SUD, investigating the course and timing of neurocognitive and psychosocial factors, including recovery (Hagen et al., 2016; Svendsen et al., 2017). In agreement with previous research on the STAYER sample (Erga et al., 2020), “polysubstance use disorder” (PSUD) refers to the use of multiple substances as part of a pattern of problematic substance use, in which the participants meet the criteria for SUD for some, but not necessarily all substances used. Nonetheless, polysubstance use is complex phenomenon and that research has defined it differently according to simultaneous or sequential use and short and long time frames (Ellis et al., 2023; Fitzgerald et al., 2022; Liu et al., 2018; Mefodeva et al., 2022). However, most researchers would agree on a consensus definition of it involving consumption of multiple substances within a specified time frame.

Previous research and reviews have found that supportive friendships in recovery networks, such as Alcoholics Anonymous or non-drug-using social networks, are related to sustained abstinence (Best et al., 2016; Drake et al., 2008; Nesvåg & McKay, 2018; Weisner et al., 2003) and reduced relapse risk (Ness et al., 2014; Nordfjaern, 2011), but the role of supportive friendships in recovery is complicated and broad. Research indicates that supportive friendships may have a positive impact on recovery, but it varies and depends on several factors such as the specificity of the measure (general or specific support to substance use), and other factors, e.g., readiness to change and age (Groh et al., 2007; McKay et al., 2011; McKay et al., 2013a). Friendships or networks that support abstinence are favorable to sustained abstinence (Litt et al., 2009). However, the support might be indirect through environmental factors (i.e., others in one's network misuse substances), rather than supporting substance use (Groh et al., 2007). Overall, previous research finds that social factors may be positive, negative or mixed in promoting recovery (McCrady, 2004).

Weisner et al. (2003) investigated to what extent individual, treatment, or extra-treatment characteristics were predictive of abstinence at the 5-year follow-up based on participants’ interviews at baseline, at 6 months and at the 5-year follow-up. They found that abstinence at 6 months predicted abstinence at 5 years, and predictors of abstinence were, among others, *being female* and *involvement in recovery-oriented social networks*. However, they might have missed other association since they did not conduct annual interviews. A systematic review found that having supportive friendships was indispensable to recovery (Vigdal et al., 2022), while a literature review found that having unsupportive drug-free friendships negatively affected recovery (Groh et al., 2008). Dennis et al. (2007) interviewed patients in treatment for alcohol or drug use about their perceived social support and number of drug-free friends at 6-month follow-up and annually for 8 years. They found a positive association between the duration of abstinence, experiencing social support and having drug-free friends, and that females were significantly more likely to maintain abstinence than men. However, the assessment of drug and alcohol use was conducted retrospectively. Thus, previous research has long intervals between participant assessments and retrospective calculations of drug and alcohol use, which include methodological uncertainties that our research aims to mitigate.

There are few Scandinavian studies of patients with SUD with comparable follow-up design to the current study. Several of the longitudinal studies are of high-risk groups, such as the Norwegian Offender Mental Health and Addiction study (NorMA) (Bukten et al., 2015), adolescents (12–16 years of age) with SUD (The Monitoring Young Lifestyles (MyLife)) (Brunborg et al., 2019) or general population based longitudinal studies such as the Norwegian Tracking Opportunities and Problems Study (Nilsen et al., 2017), resulting in issues with generalisation to patients with SUD. This is also the case for research from Finland and Sweden focusing on the general population (Nilsson et al., 2014; Savage et al., 2018), a collaboration between Norway, Denmark, Finland, Iceland and Sweden focusing on adolescents (Raitasalo et al., 2024), and The Danish longitudinal study of alcoholism 1978–2008 (Knop, 2011). In one Norwegian study with 10-year follow-up of patients with SUDs, the role of friendships for recovery trajectories where not explored (Melberg et al., 2003).

The ability to recover may also depend on substance use debut age, biological sex and gender. Research shows that obtaining recovery and reduction in substance use are associated with onset age (Dennis et al., 2005; Grella et al., 2003; Hser et al., 2007; Scott et al., 2005; Simpson et al., 1997). Females tend to have a lower risk of relapse after recovery than males (Grella et al., 2008), but gender-associated relapse risk depends on the substance used (Riley et al., 2018; Zakiniaeiz & Potenza, 2018). Although recent research suggests that females and males do not differ considerably in terms of SUD treatment outcome, the findings on biological sex and gender differences in SUDs are complicated due to the complex interaction between biological and environmental aspects (McHugh et al., 2018).

However, previous and recent reviews suggest that most SUD research is short-term and focuses on substance use reduction rather than functioning (Moe et al., 2021; Tiffany et al., 2012). Systematic reviews suggest there are few longitudinal SUD studies on drug-free relationships, debut age and gender with a duration extending 2 years (Bjornestad et al., 2020; Vanderplasschen & Best, 2021). It seems that the field needs longitudinal SUD research extending two years focusing on functional aspects and drug-free relationships of addiction recovery, and how they relate to gender, as well as substance use debut age. The present study aims to mitigate this knowledge gap by analysing changes in trajectories of drug-free friendships and alcohol and substance use, and debut age and gender (differences between female and male) annually across 4 years.

## Methods

### Sample

We recruited the study sample (*n* = 208) from the ongoing Norwegian Stavanger Study of Trajectories in Addiction (STAYER), comprising a prospective naturalistic follow-up study of change trajectories among people diagnosed with SUD investigating the course and timing of neurocognitive and psychosocial factors, including recovery (Hagen et al., 2016; Svendsen et al., 2017). Participants were included between March 2012 and December 2015, and they were recruited at the start of treatment in outpatient or residential treatment facilities in the Stavanger region of Norway. The sample consisted of patients with SUD, alcohol dependence and behavioural addictions. The STAYER study has been approved by the Regional Ethical Committee (REK 2011/1877). All participants provided written informed consent.

We included participants (1) starting a new treatment sequence within addiction treatment services; (2) aged ≥ 16 years; (3) enrolled in a treatment programme to which they were admitted for at least 2 weeks; and (4) reporting polysubstance use (i.e., patients with SUD who informed the use of multiple substances within the last year before inclusion). In Norway, patients need to have a SUD diagnosis to access specialised addiction treatment. The diagnostic assessment is carried out either by a clinical psychologist or physician and based on the criteria in the International Classification of Diseases, 10th edition (ICD-10). Of the 208 participants in the STAYER study, 164 met these criteria and were included. Due to missing data, only 155 were included in most analyses (detailed below). Details on the STAYER study methodology and retention are published elsewhere (Svendsen et al., 2017).

### Measures

Age was calculated by subtracting birth year from inclusion year at baseline. Gender was reported at baseline. Age and gender were reported in descriptive statistics but were not used in the latent growth curve analyses. *Debut age* refers to age of initiation of substance use. Because there is no defined cut-off age to indicate early versus late debut age, we chose to analyse drug debut before the age of 13 years versus debut at 13 years and older. We assessed it as favourable to divide between children and adolescents and the transition from elementary school to secondary school often follows the age of 13 years in Norway. More details on distribution of age, gender and substance use disorder are provided in Tables 1 and 2.

**Table 1.** Participants drug preferences divided by type at baseline.

Type of drug	*n* (total of 164)	Percentage
Multiple drugs	48	29.3%
Hashish/cannabis	29	17.7%
Stimulants	26	15.9%
Opiates	15	9.1%
Alcohol	8	4.9%
Benzodiazepine	4	2.4%
Gamma-hydroxybutyrate (i.e., GHB)	2	1.2%
Others	1	0.6%
Did not answer	31	18.9%

**Table 2.** Age and sex, numbers (percentage).

Age group (years)	Male, *n* (%)	Female, *n* (%)	Total, *n* (%)
Up to 18	2 (1.87%)	4 (7.02%)	6 (3.66%)
19–21	18 (16.82%)	10 (17.54%)	28 (17.07%)
22–24	23 (21.50%)	17 (29.82%)	40 (24.39%)
25–27	13 (12.15%)	11 (19.30%)	24 (14.63%)
28–30	12 (11.21%)	5 (8.77%)	17 (10.37%)
31–33	15 (14.02%)	5 (8.77%)	20 (12.20%)
34–36	5 (4.67%)	1 (1.75%)	6 (3.66%)
37–51	19 (17.76%)	4 (7.02%)	23 (14.02%)
Total	107	57	164

#### Drug and alcohol use

Drug and alcohol use was assessed using adjusted versions of the Drug Use Disorders Identification Test (DUDIT) (Voluse et al., 2012) and the Alcohol Identification Disorder Test (AUDIT) (Babor et al., 1992; Berman et al., 2005). Both DUDIT and AUDIT have been found to have good reliability and validity (Bohn et al., 1995; Hildebrand, 2015; Meneses-Gaya et al., 2009; Voluse et al., 2012). We used DUDIT-C, which consists of the four consumption items measuring drug consumption, to measure drug use (Basedow et al., 2021; Berman et al., 2005). We used AUDIT-C, which consists of the three consumption items of AUDIT, to measure alcohol use (Campbell & Maisto, 2018).

We used DUDIT-C and AUDIT-C scales (ranging from 0 to 8 and from 0 to 12), merging AUDIT-C and DUDIT-C by adding them together after dividing DUDIT-C scores by 8 and AUDIT-C scores by 12. Further, we multiplied the result by four and rounded the result to whole numbers, making a scale from zero (no drug and no alcohol) to eight (maximum on both DUDIT-C and AUDIT-C scales). This new composite variable was termed “alcohol and drug use”. For participants missing AUDIT-C, we used only DUDIT-C, and vice versa. Participants with missing scores on both AUDIT-C and DUDIT-C were coded as missing at that timepoint. We calculated “alcohol and drug use” for 5-yearly follow-ups. Previous research suggests that early treatment response measured at first follow-up is a good predictor of long-term treatment response (McKay et al., 2005; McKay et al., 2013b). Therefore, we excluded baseline measures from the latent growth models.

#### Drug-free friendships

“Drug-free friendships” was assessed using a self-report questionnaire (KVARUS) to measure social support. “Drug-free friendships” has previously been used to measure social resources (Carlsen et al., 2020). Having drug-free friendships was measured using a dichotomous question (YES/NO) at baseline and follow-ups: “Do you have friendships without a history of substance use?”

### Data analysis

Descriptive statistics, data preparation and export were computed using Stata/IC 15.1 (https://www.stata.com). Mplus, version 8 (https://www.statmodel.com) was used for the latent growth curve models (LGM). We defined the “alcohol and drug use” scale as continuous variables and used the standard ML estimator in Mplus. Because the “drug-free friendships” measures were categorical, we used the Mplus standard for categorical dependent variables: the WLSMV-estimator (Brown, 2015). To evaluate the goodness of fit for the tested models, we used the root mean square error of approximation (RMSEA) and comparative fit index (CFI). Both measures indicate the degree to which a model fits data. CFI scores closer to 1 and RMSEA scores closer to 0 indicate better model fit (Bollen & Curran, 2006). More specifically, CFI scores ≥0.95 and RMSEA scores ≤0.05 indicate good model fit (Barbara, 2012), whereas RMSEA scores between .05 and .08 have been deemed acceptable (Kim et al., 2016).

First, we examined the association between “alcohol and drug use” and “drug-free friendships” across the last five follow-ups (Table 3). We tested if these associations were significant using chi-squared tests. Second, we ran multiple longitudinal models to examine possible models for understanding the association between “alcohol and drug use” and “drug-free friendships”. We developed three LGMs. Model 1 (M1) investigated to which degree the LGM on longitudinal development in alcohol and drug use fits the data. Model fit indicators for all models were summarised in Table 4.

**Table 3.** “Alcohol and drug use” (0–8) across drug-free friendships, five waves.

		Drug-free friendships									
		1 year		2 years		3 years		4 years*		5 years	
		No	Yes	No	Yes	No	Yes	No	Yes	No	Yes
		*n* (%)	*n* (%)	*n* (%)	*n* (%)	*n* (%)	*n* (%)	*n* (%)	*n* (%)	*n* (%)	*n* (%)
“Alcohol and drug use”	0	7 (31.8)	48 (39.0)	6 (33.3)	39 (36.1)	3 (15.0)	29 (29.6)	2 (15.4)	32 (36.0)	4 (22.2)	26 (37.7)
	1	2 (9.1)	12 (9.8)	0 (0)	15 (13.9)	2 (10.0)	13 (13.3)	0 (0)	11 (12.4)	1 (5.6)	12 (17.4)
	2	4 (18.2)	11 (8.9)	1 (5.6)	12 (11.1)	1 (5.0)	18 (18.4)	4 (30.1)	17 (19.1)	3 (16.7)	12 (17.4)
	3	0 (0)	16 (13.0)	2 (11.1)	11 (10.2)	2 (10.0)	8 (8.2)	1 (7.7)	5 (5.6)	0 (0)	4 (5.8)
	4	3 (13.6)	10 (8.1)	6 (33.3)	8 (7.4)	5 (25.0)	13 (13.3)	5 (38.5)	5 (5.6)	2 (11.1)	3 (4.4)
	5	3 (13.6)	14 (11.4)	0 (0)	10 (9.3)	1 (5.0)	2 (2.0)	0 (0)	8 (9.0)	3 (16.7)	2 (2.9)
	6	2 (9.1)	8 (6.5)	2 (11.1)	7 (6.5)	2 (10.0)	3 (3.1)	0 (0)	4 (4.5)	1 (5.6)	1 (1.5)
	7	(0)	2 (1.6)	1 (5.6)	5 (4.6)	2 (10.0)	7 (7.1)	0 (0)	1 (1.1)	2 (11.1)	6 (8.7)
	8	1 (4.6)	2 (1.6)	0 (0)	1 (0.9)	2 (10.0)	5 (5.1)	1 (7.7)	6 (6.7)	2 (11.1)	3 (4.4)
	Total	22 (15.2)	123 (84.8)	18 (14.3)	108 (85.7)	20 (17.0)	98 (83.1)	13 (12.8)	89 (87.3)	18 (20.7)	69 (79.3)
	*p*	χ^2 ^= 6.69, df = 8, *p* > .05		χ^2 ^= 14.67, df = 8, *p* > .05		χ^2 ^= 8.16, df = 8, *p* > .05		χ^2 ^= 18.27, df = 8, *p* < .05		χ^2 ^= 11.49, df = 8, *p* > .05	

*Significant association.

**Table 4.** Fit indices for the tested models.

Model	*n*	χ^2^**	df**	CFI	RMSEA
M1: LGM* AUDIT-DUDIT	155	17.43	10	0.963	0.069
M2: LGM* Drug-free friends	155	2.16	6	1.000	0.000
M2 revised: LGM* Drug-free friends, intercept only	155	15.23	9	0.926	0.067
M3: Combined M1 revised and M2: two parallel LGMs: AUDIT-DUDIT and ‘drug-free friends’ (Figure 1)	155	43.33	39	0.982	0.027

*LGM = latent growth curve model.

**Chi-squared test of model fit.

AUDIT = Alcohol Identification Disorder Test; CFI = comparative fit index; DUDIT = Drug Use Disorders Identification Test; RMSEA = root mean square error of approximation.

We divided M1 into two groups based on gender. As the unconstrained model was not significantly better than the constrained model (χ^2^ difference = 11.32, df difference = 8, *p* = .18), we rejected gender groups in M1. Furthermore, we divided M1 into two groups based on drug debut before the age of 13 years versus debut at 13 years and older. The unconstrained model was not significantly better than the constrained model (χ^2^ difference = 8.31, df difference = 8, *p* = .40). Hence, we kept M1 without groups (M1). However, because participants were selected based on alcohol and drug use at baseline, we removed baseline from the LGM and used this LGM as a predictor (M1 revised).

Model 2 (M2) investigated the degree to which an LGM fits the development of “drug-free friendships”. The model was an LGM with two latent variables (intercept and slope) reflecting “drug-free friendships” development based on five dichotomous variables. Hence, the model “drug-free friendships” had too few degrees of freedom to estimate model fit. Furthermore, the variance on the slope was not significant. We therefore made a new model with intercept only (M2 revised). We chose to use the initial model (M2) because M2 fits conceptually better with “M1 revised” when making model 3 (M3).

**Figure 1.**
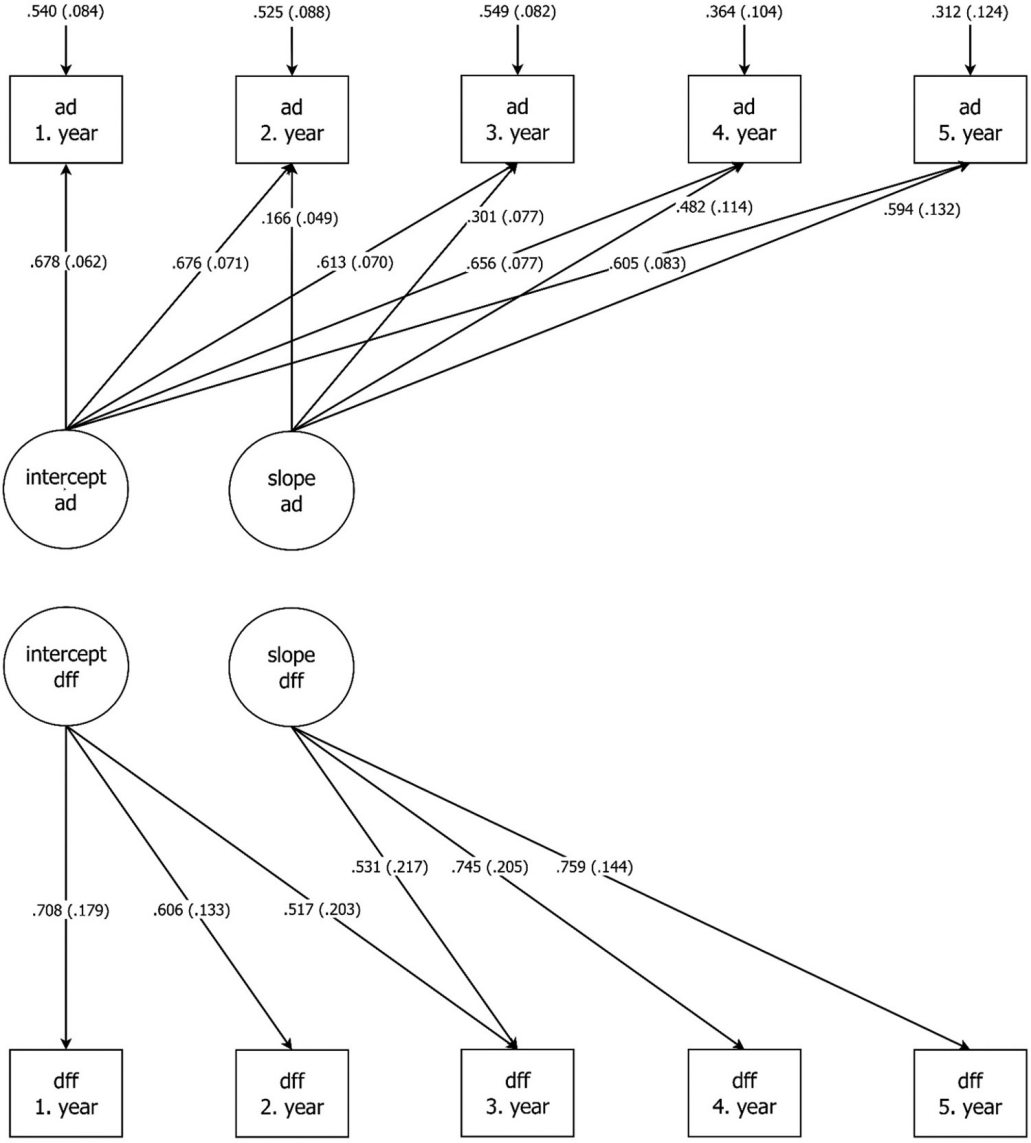
AUDIT-DUDIT (ad) and Drug-free friends (aff) latent growth models; only significant standardised path visible. AUDIT = Alcohol Identification Disorder Test; DUDIT = Drug Use Disorders Identification Test.

M3 was constructed by combining M1 revised and M2. Thus, M3 was a growth model for two parallel processes with categorical outcomes, “alcohol and drug use” and “drug-free friendships”. We allowed association between the two intercepts. Furthermore, we added a regression from the “alcohol and drug use” intercept to the slope on “drug-free friendships”. We also added a regression from the “drug-free friendships” intercept to the slope on “alcohol and drug use”. Finally, we made a figure for the final model, M3, reporting only significant and standardised weights (Figure 1).

## Results

Cross-sectional analysis displays a stable pattern of “drug-free friendships” across follow-ups, ranging from 79.3% at 5 years to 87.3% at 4 years (Table 3). Among participants having “drug-free friendships” after 4 years, 36.0% had an “alcohol and drug use” score of 0 in the same year. By contrast, 15.4% of participants without “drug-free friendships” had an “alcohol and drug use” score of 0 (χ^2^ = 18.27, df = 8, *p* < .05) (Table 3). In general, there was a clear tendency of lower “alcohol and drug use” scores for participants having “drug-free friendships” after 4 years (χ^2^ = 18.27, df = 8, *p* < .05) (Table 3). However, four of the five included annual follow-ups showed no significant difference in “alcohol and drug use” score across having “drug-free friendships” (Table 3).

The initial LGM, including five follow-ups of “alcohol and drug use”, had an acceptable fit (M1) (Table 4) and showed significant variance on both the intercept (2.42, *p* < .001) and the slope (0.501, *p* < .05), indicating small interindividual differences in the initial status of “alcohol and drug use” and the change in status over follow-ups. However, only the intercept had a significant mean (2.329, *p* < .001) and the slope mean was 0 (0.000), implying no change in the scores across follow-ups.

The initial LGM on five follow-ups of “drug-free friendships” could not calculate fit due to too few degrees of freedom (M2) (Table 4). M2 had significant variance on intercept (0.652, *p* < .05), but not on slope (0.084, *p* = .404), implying no change across follow-ups. As presented in the method section, this model was kept despite non-significant variance on the slope.

The final model, M3, had a very good fit (Figure 1 and Table 4). We did not find any significant regression path between the “alcohol and drug use” and “drug-free friendships” slope (Figure 1). As previously described in the method section, we allowed the two intercepts to be associated. However, neither this association, nor the allowed association between the slopes was significant.

## Discussion

The main finding is the stability in alcohol and drug use across 4 years (i.e., from first to fifth follow-up). Further, drug-free friendships were fairly constant across 4 years and did not affect alcohol and drug use in four of the five follow-ups.

### Stability rather than a change in alcohol and drug use

The first model analysing change trajectories in alcohol and drug use showed variations regarding a positive initial alcohol and drug use level. Regarding this development, there is still variation, but the variation is smaller with regard to the regular use of alcohol and drugs. Our finding is contrary to previous research, which often finds several different (e.g., three to four) alcohol and drug use trajectories (Hser et al., 2007; Maisto et al., 2020; Timko et al., 2016; Witkiewitz et al., 2019).

Contrary to our finding, previous studies with follow-up (e.g., 3–4 years) often find a reduction in alcohol or drug use (Dennis & Scott, 2012; McKay & Weiss, 2001; Project MATCH Research Group, 1998). According to Heyman (2013), most people suffering from addiction eventually remit. Although remittance depends on the substance to which one is addicted, Heyman concludes that if the general age of onset is 20 years, most people suffering from addiction are remitted by age 30 years. The mean age of our sample is below 30 years, and it may therefore be possible that substance use levels would have decreased if we had analysed our sample in different age groups or at a later stage during the course. However, other studies suggest that the median time from first to last use of a substance (i.e., obtaining 12 months of abstinence) is 27 years (Dennis et al., 2005).

### Drug-free friendships

Our results showed one significant association between alcohol and drug use and drug-free friendship at the fourth year (Table 3). This appears to be contrary to the vast literature on the positive association between social network involvement, social support and recovery (Ellis et al., 2004; McKay, 2017; Ness et al., 2014; Nesvåg & McKay, 2018; Nordfjaern, 2011; van Melick et al., 2013; Vigdal et al., 2022; Weisner et al., 2003). Research suggests either a positive, a negative or mixed association between drug-free relationships and recovery (McCrady, 2004), whereas we mainly found no association between alcohol and drug use, on the one hand, and drug-free friendships, on the other. Thus, the participants in our study may possibly have neither positive, nor negative drug-free friendships, which might explain why the friendships did not influence their alcohol or drug use. However, it may also reflect that the measure used in our study is not sensitive enough to capture this association.

Generally, positive drug-free friendships facilitate recovery maintenance (Lookatch et al., 2019), whereas having unsupportive drug-free friends curtails recovery (Dennis et al., 2007; Groh et al., 2008). In this respect, we may question the quality of the participants’ drug-free friendships in our study. The drug-free friendships reported may have been formed long before participants entered this study. When they were formed, the friendships may have been positive or negative, but the participants did not spend any or much time with them during follow-up. Perchance drug-free friendships may not influence the participants’ alcohol and drug use because they did not contribute any social support (nor were unsupportive). However, taking the abovementioned research into account, it seems very surprising that drug-free friendships, whether positive or negative, only influenced one of the five follow-ups on alcohol and drug use. If these friendships were actively present in these individuals’ life, they would presumably influence alcohol or drug use to some extent.

### Gender and debut age

We found that neither gender, nor debut age had any significant association with drug use trajectories. Our result is contrary to previous research, showing that age of onset prior to 21 years, particularly prior to 15 years, is associated with higher substance use levels compared to people with higher onset age (Dennis et al., 2005; Poudel & Gautam, 2017; Scott et al., 2005). However, our sample size is smaller compared to the three previous studies and have a different research design which may explain the difference. Contrary to previous research (Grella et al., 2003; Hser et al., 2007; Simpson et al., 1997), our findings suggest that debut age may not reduce the possibility of recovery because it is not associated with alcohol and drug use trajectories. Studies show that early onset of substance use is a strong predictor of later regular use (Woodcock et al., 2015) and correlated not only with adverse health-related outcomes such as adolescent suicide, but also challenges at school (Piehler et al., 2012). Additionally, early onset has been related to a greater level of family deviance and deviant behaviour (Gordon et al., 2004) and experiencing childhood trauma has been associated with alcohol and drug use challenges (Mandavia et al., 2016). In this context, our findings warrant more research before firm conclusions can be drawn.

In terms of gender, our result implies that gender may not influence alcohol and drug use trajectories, thus indicating equal opportunities for males and females to achieve recovery. Our finding is contrary to previous research, which shows that women tend to have lower risks of relapse after recovery than men (Grella et al., 2008) and that there is a gender difference in substance use levels (Riley et al., 2018; Salom et al., 2016; Zakiniaeiz & Potenza, 2018). Recent studies on gender differences indicate different recovery mechanisms and mediators for women compared to men (Andersson et al., 2021). Thus, we would have expected a difference in alcohol and drug use trajectories between men and women in our study.

### Implications of results

The stability in alcohol and drug use trajectories may suggest a need for long-term follow-up to reduce alcohol and drug use gradually over several years. This seems to be in agreement with previous research showing that alcohol and drug use reduction and abstinence takes many years (Dennis & Scott, 2007; Dennis et al., 2005; Heyman, 2013).

Our findings suggest that interventions other than drug-free friendships may be more relevant to facilitating recovery, such as employment or social networks (Ellis et al., 2004; McKay, 2017; Ness et al., 2014; Nesvåg & McKay, 2018; Nordfjaern, 2011; van Melick et al., 2013; Vigdal et al., 2022; Weisner et al., 2003). However, the literature also suggests that drug-free friendships may be positive, negative or mixed (McCrady, 2004). More research than our study is needed before any definite conclusion on excluding drug-free friendships can be drawn, insofar as our results seem counterintuitive given previous research showing positive effects (see limitations). We suggest a similar conclusion for our results on debut age and gender. Studies suggest a relationship between gender and recovery, and that debut age is associated with adverse health-related and social outcomes.

The stability in alcohol and drug use trajectories across 4 years suggests the need for more longitudinal research. It may be warranted to investigate the frequency and quality of drug-free friendships, participant friendship assessment, including what study participants associate with having a friend, and also whether the friendships are positive, negative or both to participants’ recovery.

### Strengths and limitations

We consider it a strength that our study is one of the few longitudinal studies with social variables, gender and PSUD. However, the current understanding of gender identities suggests there are more than two genders. Because we only investigated the difference between two genders, we might have missed other gender differences. Our alcohol and drug use measures only yielded sum scores, which may be regarded as a limitation. Further, the DUDIT-C may be regarded as a crude measure of polysubstance use. The variable “drug-free friendships” is a dichotomous variable and does not yield information about the frequency and quality of drug-free friendships and participants’ friendship assessments. Thus, the measure may not be sufficiently sensitive, which may be indicated by the high scores in the cross-sectional analysis (Table 3). Further, the findings are based on a small dataset, and perhaps a more extensive dataset would yield one or more significant associations between SUD and drug-free friendships. For example, small samples have larger standard error estimates which affects the accuracy of statistical inferences. However, small sample size is a common challenge with longitudinal research (McNeish, 2019). Statistical methods were chosen on model complexity, sample size and research questions. The results may also be due to little variation in SUD and drug-free friendships, both at initial levels and in development. Further, a skewed age distribution towards younger people may be part of the reason why our findings differ from previous research. We have limited information about the participants’ diagnostic assessment and thus we have limited information about number of SUDs and the types of SUD they have. Our assessment of participants’ PSUD is based on their AUDIT and DUDIT scores showing that they use more than one substance. However, our assessment is not based on the initial diagnostic evaluation, which is a limitation. Lastly, we did not examine to which degree treatment (duration, amount, intensity) may have influenced the participants alcohol and drug use, which is a shortcoming.

## References

[bibr1-14550725251332929] Andersson C. Wincup E. Best D. Irving J. (2021). Gender and recovery pathways in the UK. Drugs: Education, Prevention and Policy, 28(5), 454–464. 10.1080/09687637.2020.1852180

[bibr2-14550725251332929] Babor T. F. De La Fuente J. R. Saunders J. Grant M. (1992). AUDIT. The Alcohol Use Disorders Identification Test: Guidelines for use in primary health care. World Health Organization.

[bibr3-14550725251332929] Barbara M. (2012). Structural equation modeling with Mplus: basic concepts, application, and programming. Taylor & Francis Group.

[bibr4-14550725251332929] Basedow L. A. Kuitunen-Paul S. Eichler A. Roessner V. Golub Y. (2021). Diagnostic accuracy of the drug use disorder identification test and its short form, the DUDIT-C, in German adolescent psychiatric patients. Frontiers in Psychology, 12(6), 678819–678819. 10.3389/fpsyg.2021.678819 34149570 PMC8212997

[bibr5-14550725251332929] Berman A. H. Bergman H. Palmstierna T. Schlyter F. (2005). Evaluation of the drug use disorders identification test (DUDIT) in criminal justice and detoxification settings and in a Swedish population sample. European Addiction Research, 11(1), 22–31. 10.1159/000081413 15608468

[bibr6-14550725251332929] Best D. Beckwith M. Haslam C. Alexander Haslam S. Jetten J. Mawson E. Lubman D. I. (2016). Overcoming alcohol and other drug addiction as a process of social identity transition: The social identity model of recovery (SIMOR). Addiction Research & Theory, 24(2), 111–123. 10.3109/16066359.2015.1075980

[bibr7-14550725251332929] Bhalla I. P. Stefanovics E. A. Rosenheck R. A. (2017). Clinical epidemiology of single versus multiple substance use disorders: Polysubstance use disorder. Medical Care, 55(9), S24–S32. 10.1097/MLR.0000000000000731 28806363

[bibr8-14550725251332929] Bjornestad J. McKay J. R. Berg H. Moltu C. Nesvåg S. (2020). How often are outcomes other than change in substance use measured? A systematic review of outcome measures in contemporary randomised controlled trials. Drug and Alcohol Review, 39(4), 394–414. 10.1111/dar.13051 32147903

[bibr9-14550725251332929] Bohn M. J. Babor T. F. Kranzler H. R. (1995). The Alcohol Use Disorders Identification Test (AUDIT): Validation of a screening instrument for use in medical settings. Journal of Studies on Alcohol, 56(4), 423–432. 10.15288/jsa.1995.56.423 7674678

[bibr10-14550725251332929] Bollen K. A. Curran P. J. (2006). Latent curve models: A structural equation perspective. John Wiley & Sons.

[bibr11-14550725251332929] Brown T. A. (2015). Confirmatory factor analysis for applied research. The Guilford Press.

[bibr12-14550725251332929] Brunborg G. S. Scheffels J. Tokle R. Buvik K. Kvaavik E. Burdzovic Andreas J. (2019). Monitoring young lifestyles (MyLife) - a prospective longitudinal quantitative and qualitative study of youth development and substance use in Norway. BMJ Open, 9(10), e031084. 10.1136/bmjopen-2019-031084 PMC6830719 31662382

[bibr13-14550725251332929] Bukten A. Lund I. O. Rognli E. B. Stavseth M. R. Lobmaier P. Skurtveit S. Clausen T. Kunøe N. (2015). The Norwegian offender mental health and addiction study–design and implementation of a national survey and prospective cohort study. Substance Abuse: Research and Treatment, 9(11), SART. S23546. 10.4137/SART.S23546 PMC4666526 26648732

[bibr14-14550725251332929] Campbell C. E. Maisto S. A. (2018). Validity of the AUDIT-C screen for at-risk drinking among students utilizing university primary care. Journal of American College Health: J of ACH, 66(8), 774–782. 10.1080/07448481.2018.1453514 PMC6151161 29565778

[bibr15-14550725251332929] Carlsen S.-E. L. Lunde L.-H. Torsheim T. (2020). Opioid and polydrug use among patients in opioid maintenance treatment. Substance Abuse and Rehabilitation, 11(1), 9. 10.2147/SAR.S221618 32099510 PMC6996215

[bibr16-14550725251332929] Davidson L. Tondora J. O'Connell M. J. Kirk T. Jr., Rockholz P. Evans A. C. (2007). Creating a recovery-oriented system of behavioral health care: Moving from concept to reality. Psychiatric Rehabilitation Journal, 31(1), 23. 10.2975/31.1.2007.23.31 17694712

[bibr17-14550725251332929] Dennis M. L. Foss M. A. Scott C. K. (2007). An eight-year perspective on the relationship between the duration of abstinence and other aspects of recovery. Evaluation Review, 31(6), 585–612. 10.1177/0193841X07307771 17986709

[bibr18-14550725251332929] Dennis M. Scott C. K. (2007). Managing addiction as a chronic condition. Addiction Science & Clinical Practice, 4(1), 45. 10.1151/ascp074145 18292710 PMC2797101

[bibr19-14550725251332929] Dennis M. L. Scott C. K. (2012). Four-year outcomes from the early Re-intervention (ERI) experiment using recovery management checkups (RMCs). Drug & Alcohol Dependence, 121(1–2), 10–17. 10.1016/j.drugalcdep.2011.07.026 21903347 PMC3277866

[bibr20-14550725251332929] Dennis M. L. Scott C. K. Funk R. Foss M. A. (2005). The duration and correlates of addiction and treatment careers. Journal of Substance Abuse Treatment, 28(2), S51–S62. 10.1016/j.jsat.2004.10.013 15797639

[bibr21-14550725251332929] Drake R. E. O'Neal E. L. Wallach M. A. (2008). A systematic review of psychosocial research on psychosocial interventions for people with co-occurring severe mental and substance use disorders. Journal of Substance Abuse Treatment, 34(1), 123–138. 10.1016/j.jsat.2007.01.011 17574803

[bibr22-14550725251332929] Ellis B. Bernichon T. Yu P. Roberts T. Herrell J. M. (2004). Effect of social support on substance abuse relapse in a residential treatment setting for women. Evaluation and Program Planning, 27(2), 213–221. 10.1016/j.evalprogplan.2004.01.011

[bibr23-14550725251332929] Ellis J. D. Rabinowitz J. A. Ware O. D. Wells J. Dunn K. E. Huhn A. S. (2023). Patterns of polysubstance use and clinical comorbidity among persons seeking substance use treatment: An observational study. Journal of Substance Use and Addiction Treatment, 146(1), 208932. 10.1016/j.josat.2022.208932 36880895 PMC10035066

[bibr24-14550725251332929] Erga A. H. Honsi A. Anda-Agotnes L. G. Nesvag S. Hesse M. Hagen E. (2020). Trajectories of psychological distress during recovery from polysubstance use disorder. Addiction Research & Theory, 29(1), 64–71. 10.1080/16066359.2020.1730822

[bibr25-14550725251332929] Fitzgerald N. D. Liu Y. Wang A. Striley C. W. Setlow B. Knackstedt L. Cottler L. B. (2022). Test-retest reliability of a new assessment to detect detailed temporal patterns of polysubstance use. International Journal of Methods in Psychiatric Research, 31(3), e1912. 10.1002/mpr.1912 PMC9464326 35684977

[bibr26-14550725251332929] Fowler D. Hodgekins J. French P. (2019). Social Recovery Therapy in improving activity and social outcomes in early psychosis: Current evidence and longer term outcomes. Schizophrenia Research, 203(1), 99–104. 10.1016/j.schres.2017.10.006 29070442 PMC6336979

[bibr27-14550725251332929] Gordon M. S. Kinlock T. W. Battjes R. J. (2004). Correlates of early substance use and crime among adolescents entering outpatient substance abuse treatment. American Journal of Drug and Alcohol Abuse, 30(1), 39–59. 10.1081/ada-120029865 15083553

[bibr28-14550725251332929] Grella C. E. Hser Y.-I. Hsieh S.-C. (2003). Predictors of drug treatment re-entry following relapse to cocaine use in DATOS. Journal of Substance Abuse Treatment, 25(3), 145–154. 10.1016/S0740-5472(03)00128-4 14670520

[bibr29-14550725251332929] Grella C. E. Scott C. K. Foss M. A. Dennis M. L. (2008). Gender similarities and differences in the treatment, relapse, and recovery cycle. Evaluation Review, 32(1), 113–137. 10.1177/0193841X07307318 18198172 PMC3025819

[bibr30-14550725251332929] Groh D. R. Jason L. A. Keys C. B. (2008). Social network variables in alcoholics anonymous: A literature review. Clinical Psychology Review, 28(3), 430–450. 10.1016/j.cpr.2007.07.014 17719158 PMC2289871

[bibr31-14550725251332929] Groh D. R. Olson B. D. Jason L. A. Davis M. I. Ferrari J. R. (2007). A factor analysis of the important people inventory. Alcohol & Alcoholism, 42(4), 347–353. 10.1093/alcalc/agm012 17510103 PMC3014731

[bibr32-14550725251332929] Hagen E. Erga A. H. Hagen K. P. Nesvåg S. M. McKay J. R. Lundervold A. J. Walderhaug E. (2016). Assessment of executive function in patients with substance use disorder: A comparison of inventory-and performance-based assessment. Journal of Substance Abuse Treatment, 66(7), 1–8. 10.1016/j.jsat.2016.02.010 27211990

[bibr33-14550725251332929] Hasin D. S. O’brien C. P. Auriacombe M. Borges G. Bucholz K. Budney A. Compton W. M. Crowley T. Ling W. Petry N. M. (2013). DSM-5 criteria for substance use disorders: Recommendations and rationale. American Journal of Psychiatry, 170(8), 834–851. 10.1176/appi.ajp.2013.12060782 23903334 PMC3767415

[bibr34-14550725251332929] Hetland J. Braatveit K. J. Hagen E. Lundervold A. J. Erga A. H. (2021). Prevalence and characteristics of borderline intellectual functioning in a cohort of patients with polysubstance use disorder. Frontiers in Psychiatry, 12(7), 651028. 10.3389/fpsyt.2021.651028 34335320 PMC8316764

[bibr35-14550725251332929] Heyman G. M. (2013). Quitting drugs: Quantitative and qualitative features. Annual Review of Clinical Psychology, 9(1), 29–59. 10.1146/annurev-clinpsy-032511-143041 23330937

[bibr36-14550725251332929] Hildebrand M. (2015). The psychometric properties of the drug use disorders identification test (DUDIT): A review of recent research. Journal of Substance Abuse Treatment, 53(6), 52–59. 10.1016/j.jsat.2015.01.008 25682718

[bibr37-14550725251332929] Hser Y.-I. Longshore D. Anglin M. D. (2007). The life course perspective on drug use: A conceptual framework for understanding drug use trajectories. Evaluation Review, 31(6), 515–547. 10.1177/0193841X07307316 17986706

[bibr38-14550725251332929] Kim H. Ku B. Kim J. Y. Park Y.-J. Park Y.-B. (2016). Confirmatory and exploratory factor analysis for validating the phlegm pattern questionnaire for healthy subjects. Evidence-based Complementary and Alternative Medicine: ECAM, 2016(3), 2696019–2696019. 10.1155/2016/2696019 27051447 PMC4804052

[bibr39-14550725251332929] Knop J. (2011). The danish longitudinal study of alcoholism 1978-2008. Danish Medical Bulletin, 58(8), B4315. 21827728

[bibr40-14550725251332929] Leamy M. Bird V. Le Boutillier C. Williams J. Slade M. (2011). Conceptual framework for personal recovery in mental health: Systematic review and narrative synthesis. The British Journal of Psychiatry, 199(6), 445–452. 10.1192/bjp.bp.110.083733 22130746

[bibr41-14550725251332929] Liberman R. P. Kopelowicz A. Ventura J. Gutkind D. (2002). Operational criteria and factors related to recovery from schizophrenia. International Review of Psychiatry, 14(4), 256–272. 10.1080/0954026021000016905

[bibr42-14550725251332929] Litt M. D. Kadden R. M. Kabela-Cormier E. Petry N. M. (2009). Changing network support for drinking: Network support project 2-year follow-up. Journal of Consulting and Clinical Psychology, 77(2), 229–242. 10.1037/a0015252 19309183 PMC2661035

[bibr43-14550725251332929] Liu Y. Williamson V. G. Setlow B. Cottler L. B. Knackstedt L. A. (2018). The importance of considering polysubstance use: Lessons from cocaine research. Drug and Alcohol Dependence, 192(11), 16–28. 10.1016/j.drugalcdep.2018.07.025 30195242 PMC7450360

[bibr44-14550725251332929] Lookatch S. J. Wimberly A. S. McKay J. R. (2019). Effects of social support and 12-step involvement on recovery among people in continuing care for cocaine dependence. Substance Use & Misuse, 54(13), 2144–2155. 10.1080/10826084.2019.1638406 31322037 PMC6803054

[bibr45-14550725251332929] Maisto S. A. Hallgren K. A. Roos C. R. Swan J. E. Witkiewitz K. (2020). Patterns of transitions between relapse to and remission from heavy drinking over the first year after outpatient alcohol treatment and their relation to long-term outcomes. Journal of Consulting and Clinical Psychology, 88(12), 1119. 10.1037/ccp0000615 33370135 PMC7900838

[bibr46-14550725251332929] Mandavia A. Robinson G. G. N. Bradley B. Ressler K. J. Powers A. (2016). Exposure to childhood abuse and later substance use: Indirect effects of emotion dysregulation and exposure to trauma. Journal of Traumatic Stress, 29(5), 422–429. 10.1002/jts.22131 27622844 PMC5064859

[bibr47-14550725251332929] McCrady B. S. (2004). To have but one true friend: Implications for practice of research on alcohol use disorders and social network. Psychology of Addictive Behaviors, 18(2), 113–121. 10.1037/0893-164x.18.2.113 15238053

[bibr48-14550725251332929] McHugh R. K. Votaw V. R. Sugarman D. E. Greenfield S. F. (2018). Sex and gender differences in substance use disorders. Clinical Psychology Review, 66(12), 12–23. 10.1016/j.cpr.2017.10.012 29174306 PMC5945349

[bibr49-14550725251332929] McKay J. R. (2017). Making the hard work of recovery more attractive for those with substance use disorders. Addiction, 112(5), 751–757. 10.1111/add.13502 27535787 PMC5315690

[bibr50-14550725251332929] McKay J. R. Lynch K. G. Shepard D. S. Pettinati H. M. (2005). The effectiveness of telephone-based continuing care for alcohol and cocaine dependence: 24-month outcomes. Archives of General Psychiatry, 62(2), 199–207. 10.1001/archpsyc.62.2.199 15699297

[bibr51-14550725251332929] McKay J. R. Van Horn D. H. Lynch K. G. Ivey M. Cary M. S. Drapkin M. L. Coviello D. M. Plebani J. G. (2013b). An adaptive approach for identifying cocaine dependent patients who benefit from extended continuing care. Journal of Consulting and Clinical Psychology, 81(6), 1063. 10.1037/a0034265 24041231 PMC3938091

[bibr52-14550725251332929] McKay J. R. Van Horn D. Oslin D. W. Ivey M. Drapkin M. L. Coviello D. M. Yu Q. Lynch K. G. (2011). Extended telephone-based continuing care for alcohol dependence: 24-month outcomes and subgroup analyses. Addiction, 106(10), 1760–1769. 10.1111/j.1360-0443.2011.03483.x 21545667 PMC3174323

[bibr53-14550725251332929] McKay J. R. Van Horn D. Rennert L. Drapkin M. Ivey M. Koppenhaver J. (2013a). Factors in sustained recovery from cocaine dependence. Journal of Substance Abuse Treatment, 45(2), 163–172. 10.1016/j.jsat.2013.02.007 23561331 PMC3696509

[bibr54-14550725251332929] McKay J. R. Weiss R. V. (2001). A review of temporal effects and outcome predictors in substance abuse treatment studies with long-term follow-ups - preliminary results and methodological issues. Evaluation Review, 25(2), 113–161. 10.1177/0193841(0102500202 11317714

[bibr55-14550725251332929] McNeish D. (2019). Two-Level dynamic structural equation models with small samples. Structural Equation Modeling, 26(6), 948–966. 10.1080/10705511.2019.1578657 32863699 PMC7451754

[bibr56-14550725251332929] Mefodeva V. Carlyle M. Walter Z. Chan G. Hides L. (2022). Polysubstance use in young people accessing residential and day-treatment services for substance use: Substance use profiles, psychiatric comorbidity and treatment completion. Addiction, 117(12), 3110–3120. 10.1111/add.16008 35851706 PMC9804256

[bibr57-14550725251332929] Melberg H. O. Lauritzen G. O. Ravndal E. (2003). *Hvilken nytte, for hvem og til hvilken kostnad? En prospektiv studie av stoffmisbrukere i behandling* .

[bibr58-14550725251332929] Meneses-Gaya C. d. Zuardi A. W. Loureiro S. R. Crippa J. A. S. (2009). Alcohol Use Disorders Identification Test (AUDIT): An updated systematic review of psychometric properties. Psychology & Neuroscience, 2(1), 83–97. 10.3922/j.psns.2009.1.12

[bibr59-14550725251332929] Moe F. Moltu C. McKay J. Nesvaag S. Bjornestad J. (2021). Is the relapse concept in studies of substance use disorders a ‘one size fits all’ concept? A systematic review of relapse operationalisations. Drug and Alcohol Review, 41(4), 743–758. 10.1111/dar.13401 34792839

[bibr60-14550725251332929] Ness O. Borg M. Davidson L. (2014). Facilitators and barriers in dual recovery: a literature review of first-person perspectives. Advances in Dual Diagnosis, 7(3), 107–117. 10.1108/ADD-02-2014-0007

[bibr61-14550725251332929] Nesvåg S. McKay J. R. (2018). Feasibility and effects of digital interventions to support people in recovery from substance use disorders: Systematic review. Journal of Medical Internet Research, 20(8), e255. 10.2196/jmir.9873 PMC6127498 30139724

[bibr62-14550725251332929] Nilsen W. Kjeldsen A. Karevold E. B. Skipstein A. Sand Helland M. Gustavson K. Enstad F. Baardstu S. Røysamb E. von Soest T. (2017). Cohort profile: The tracking opportunities and problems study (TOPP)–study of Norwegian children and their parents followed from infancy to early adulthood. International Journal of Epidemiology, 46(5), 1399–1399g. 10.1093/ije/dyx057 28498979

[bibr63-14550725251332929] Nilsson A. Estrada F. Bäckman O. (2014). Offending, drug abuse and life chances—A longitudinal study of a Stockholm birth cohort. Journal of Scandinavian Studies in Criminology and Crime Prevention, 15(2), 128–142. 10.1080/14043858.2014.939452

[bibr64-14550725251332929] Nordfjaern T. (2011). Relapse patterns among patients with substance use disorders. Journal of Substance Use, 16(4), 313–329. 10.3109/14659890903580482

[bibr65-14550725251332929] Onyeka I. N. Uosukainen H. Korhonen M. J. Beynon C. Bell J. S. Ronkainen K. Föhr J. Tiihonen J. Kauhanen J. (2012). Sociodemographic characteristics and drug abuse patterns of treatment-seeking illicit drug abusers in Finland, 1997–2008: The huuti study. Journal of Addictive Diseases, 31(4), 350–362. 10.1080/10550887.2012.735563 23244554

[bibr66-14550725251332929] Pettersen H. Landheim A. Skeie I. Biong S. Brodahl M. Oute J. Davidson L. (2019). How social relationships influence substance use disorder recovery: A collaborative narrative study. Substance Abuse: Research and Treatment, 13(3), 1178221819833379. 10.1177/1178221819833379 30886519 PMC6410387

[bibr67-14550725251332929] Piehler T. F. Véronneau M.-H. Dishion T. J. (2012). Substance use progression from adolescence to early adulthood: Effortful control in the context of friendship influence and early-onset use. Journal of Abnormal Child Psychology, 40(7), 1045–1058. 10.1007/s10802-012-9626-7 22527607 PMC3424390

[bibr68-14550725251332929] Poudel A. Gautam S. (2017). Age of onset of substance use and psychosocial problems among individuals with substance use disorders. BMC Psychiatry, 17(1), 10. 10.1186/s12888-016-1191-0 28077106 PMC5225546

[bibr69-14550725251332929] Price-Robertson R. Obradovic A. Morgan B. (2017). Relational recovery: Beyond individualism in the recovery approach. Advances in Mental Health, 15(2), 108–120. 10.1080/18387357.2016.1243014

[bibr70-14550725251332929] Project MATCH Research Group (1998). Matching alcoholism treatments to client heterogeneity: Project MATCH three-year drinking outcomes. Alcoholism: Clinical & Experimental Research, 22(6), 1300–1311. 10.1111/j.1530-0277.1998.tb03912.x 9756046

[bibr71-14550725251332929] Raitasalo K. Rossow I. Moan I. S. Bye E. K. Svensson J. Thor S. Ekholm O. Pisinger V. Arnarsson Á Bloomfield K. (2024). Changes in co-use of alcohol and cannabis among nordic adolescents in the 21st century: Results from the European school survey project on alcohol and other drugs study. Drug and Alcohol Review, 43(3), 616–624. 10.1111/dar.13672 37095643

[bibr72-14550725251332929] Riley A. L. Hempel B. J. Clasen M. M. (2018). Sex as a biological variable: Drug use and abuse. Physiology & Behavior, 187(4), 79–96. 10.1016/j.physbeh.2017.10.005 29030249

[bibr73-14550725251332929] Salom C. L. Betts K. S. Williams G. M. Najman J. M. Alati R. (2016). Predictors of comorbid polysubstance use and mental health disorders in young adults—a latent class analysis. Addiction, 111(1), 156–164. 10.1111/add.13058 26190689

[bibr74-14550725251332929] Savage J. E. Rose R. J. Pulkkinen L. Silventoinen K. Korhonen T. Kaprio J. Gillespie N. Dick D. M. (2018). Early maturation and substance use across adolescence and young adulthood: A longitudinal study of Finnish twins. Development and Psychopathology, 30(1), 79–92. 10.1017/s0954579417000487 28424107 PMC5680125

[bibr75-14550725251332929] Scott C. K. Dennis M. L. Foss M. A. (2005). Utilizing recovery management checkups to shorten the cycle of relapse, treatment reentry, and recovery. Drug & Alcohol Dependence, 78(3), 325–338. http://search.ebscohost.com/login.aspx?direct=true&db=c8h&AN=106510714&scope=site 10.1016/j.drugalcdep.2004.12.005 15893164 PMC5933845

[bibr76-14550725251332929] Simpson D. D. Joe G. W. Brown B. S. (1997). Treatment retention and follow-up outcomes in the Drug Abuse Treatment Outcome Study (DATOS). Psychology of Addictive Behaviors, 11(4), 294. 10.1037/0893-164X.11.4.294

[bibr77-14550725251332929] Svendsen T. S. Erga A. H. Hagen E. McKay J. R. Njå A. L. M. Årstad J. Nesvåg S. (2017). How to maintain high retention rates in long-term research on addiction: A case report. Journal of Social Work Practice in the Addictions, 17(4), 374–387. 10.1080/1533256X.2017.1361831

[bibr78-14550725251332929] Tiffany S. T. Friedman L. Greenfield S. F. Hasin D. S. Jackson R. (2012). Beyond drug use: A systematic consideration of other outcomes in evaluations of treatments for substance use disorders. Addiction, 107(4), 709–718. 10.1111/j.1360-0443.2011.03581.x 21981638 PMC3257402

[bibr79-14550725251332929] Timko C. Moos R. H. Finney J. W. (2016). The course of substance use disorders: Trajectories, endpoints, and predictors. In Long-term outcomes in psychopathology research: Rethinking the scientific agenda (pp. 53–76). Oxford University Press.

[bibr80-14550725251332929] Vanderplasschen W. Best D. (2021). Mechanisms and mediators of addiction recovery. Drugs: Education, Prevention and Policy, 28(5), 385–388. 10.1080/09687637.2021.1982521

[bibr81-14550725251332929] van Melick M. McCartney D. Best D. (2013). Ongoing recovery support and peer networks: A preliminary investigation of recovery peer supporters and their peers. Journal of Groups in Addiction & Recovery, 8(3), 185–199. 10.1080/1556035X.2013.785211

[bibr82-14550725251332929] Vigdal M. I. Moltu C. Bjornestad J. Selseng L. B. (2022). Social recovery in substance use disorder: A metasynthesis of qualitative studies. Drug and Alcohol Review, 41(4), 974–987. 10.1111/dar.13434 35104369 PMC9306622

[bibr83-14550725251332929] Voluse A. C. Gioia C. J. Sobell L. C. Dum M. Sobell M. B. Simco E. R. (2012). Psychometric properties of the Drug Use Disorders Identification Test (DUDIT) with substance abusers in outpatient and residential treatment. Addictive Behaviors, 37(1), 36–41. 10.1016/j.addbeh.2011.07.030 21937169

[bibr84-14550725251332929] Weisner C. Ray G. Mertens J. R. Satre D. D. Moore C. (2003). Short-term alcohol and drug treatment outcomes predict long-term outcome [Empirical Study; Qualitative Study; Quantitative Study]. Drug and Alcohol Dependence, 71(3), 281–294. 10.1016/S0376-8716(03)00167-4 12957346

[bibr85-14550725251332929] Witkiewitz K. Montes K. S. Schwebel F. J. Tucker J. A. (2020). What is recovery? Alcohol research: Current Reviews, 40(3), 1. 10.35946/arcr.v40.3.01 PMC7505137 32983748

[bibr86-14550725251332929] Witkiewitz K. Wilson A. D. Pearson M. R. Montes K. S. Kirouac M. Roos C. R. Hallgren K. A. Maisto S. A. (2019). Profiles of recovery from alcohol use disorder at three years following treatment: Can the definition of recovery be extended to include high functioning heavy drinkers? Addiction, 114(1), 69–80. 10.1111/add.14403 30063267 PMC6289769

[bibr87-14550725251332929] Woodcock E. A. Lundahl L. H. Stoltman J. J. K. Greenwald M. K. (2015). Progression to regular heroin use: Examination of patterns, predictors, and consequences. Addictive Behaviors, 45(2), 287–293. 10.1016/j.addbeh.2015.02.014 25765913 PMC5541382

[bibr88-14550725251332929] Zakiniaeiz Y. Potenza M. N. (2018). Gender-related differences in addiction: A review of human studies. Current Opinion in Behavioral Sciences, 23(10), 171–175. 10.1016/j.cobeha.2018.08.004 39896826 PMC11784943

